# Cervical Cancer and Human Papillomavirus Awareness among Women in Saudi Arabia

**DOI:** 10.3390/medicina57121373

**Published:** 2021-12-17

**Authors:** Khalid Akkour, Lolowah Alghuson, Hicham Benabdelkamel, Hani Alhalal, Nada Alayed, Amal AlQarni, Maria Arafah

**Affiliations:** 1 Obstetrics and Gynecology Department, College of Medicine, King Saud University, Riyadh 11461, Saudi Arabia; anartist@live.com (L.A.); alhalalhani@gmail.com (H.A.); Nalayed@ksu.edu.sa (N.A.); 2 Proteomics Resource Unit, Obesity Research Center, College of Medicine, King Saud University, Riyadh 11461, Saudi Arabia; hbenabdelkamel@ksu.edu.sa; 3 College of Medicine, King Saud University, King Saud University Medical City, Riyadh 11461, Saudi Arabia; amalaliqarnii@gmail.com; 4 Department of Pathology, College of Medicine, King Saud University, King Saud University Medical City, Riyadh 11461, Saudi Arabia; marafah83@gmail.com

**Keywords:** human papillomavirus, HPV, cervical cancer, CC, HPV vaccine

## Abstract

*Background and Objectives*: Cervical cancer (CC) is the eighth most common cancer among Saudi women of all ages. With limited national data, we aimed to evaluate the public awareness of cervical cancer, CC risk factors, HPV infection, and HPV vaccines in different regions of Saudi Arabia. *Materials and Methods:* This was a survey-based cross-sectional study that encompassed 564 Saudi women over a period of a month. A self-administrated questionnaire was distributed through different social media platforms. *Results:* The collected data included sociodemographic variables and questions assessing awareness of CC, and the attitudes toward CC screening and human papillomavirus (HPV) vaccination. Most respondents were aware of CC (84.0%), although their primary source of information was the internet. However, only 45 females (8.0%) had a history of cervical screening. Furthermore, most females did not know that HPV was transmitted sexually (78.9%), or that it caused genital warts (81.7%) and CC (81.9%). Regarding the HPV vaccine, 100 females (17.7%) had heard about it, but only 11 (2.0%) took the vaccine, although more than half of the respondents (54.1%) were willing to take the vaccine after being informed about it. *Conclusions*: We noticed a remarkable lack of awareness among the respondents regarding HPV’s clinical implications; and the HPV vaccine, and its importance and availability. The main source of information for most of the Saudi women in this study was the internet, which may be an unreliable source, or provide misleading information that may delay screening or discourage vaccination. Thus, organized campaigns by the Ministry of Health or other health-advocating agencies, in addition to screening and vaccination programs, are strongly encouraged.

## 1. Introduction

Cervical cancer (CC) is one of the most common causes of gynecological cancer-related morbidity and mortality [1]. It is the fourth most prevalent malignancy in women worldwide, with an estimated 570,000 new cases and 311,000 deaths in 2018 [2]. Despite the concerns that not all CC cases were discovered in Saudi Arabia, it remains the 13th most frequent cancer among Saudi women of all ages, and the sixth in those between 15 and 44 years of age [3]. According to the Saudi Ministry of Health (MOH), the incidence rate for CC is 1.9 cases per 100,000 women in Saudi Arabia [3]. The Saudi MOH published HPV screening practice guidelines in 2014, but given the reported low incidence of CC, and from a cost effectiveness point of view, there is no national screening program for human papillomavirus (HPV) and CC in Saudi Arabia yet [3,4]. HPV is the leading causative organism of cervical cancer and cervical cancer-related mortality; women with HPV are two times more likely to gain human immunodeficiency virus (HIV) infection, and women with HIV are four times more likely to gain CC [2,5]. However, due to the discovery and implementation of HPV vaccines against high-risk serotypes, the incidence of CC has dropped in most developed countries [5,6]. Unfortunately, previous regional reports have shown that most Saudi females are oblivious to the presence of the HPV vaccine [6]. A study from the southern region showed that only 3% of non-medical female students were aware of the HPV vaccine [7]. Furthermore, a study from the western region showed that 14% of women knew that HPV is a cause of CC, and less than 10% were aware of the HPV vaccine [6]. Another study from the central region showed moderate knowledge of cervical cancer, with poor attitudes toward prevention [8]. Awareness campaigns regarding HPV exist in big cities, but are still very few; we noticed that even after the awareness campaigns, women still question the value of HPV vaccination, as they think that they do not have the same risk factors as the rest of the world due to their cultural and religious beliefs. Nation-wide data regarding the prevalence of CC or abnormal pap smears, as well as the awareness or implementation of the HPV vaccine, are limited for multiple reasons; for example, access to a health system for cancer cases is not always easy, and there is a lack of national screening programs where CC cases can be discovered in their early stages. Furthermore, not all advanced cervical cancer cases are registered; the patient might die before proper diagnosis, as one of the major health issues in Saudi Arabia is the late presentation of cancer cases, and the culture barriers against postmortem diagnostic autopsies [9,10]. Unfortunately, most women in developing countries, including Saudi Arabia, present with clinically advanced stages requiring extensive survival treatment, including surgery, radiotherapy, and chemotherapy [11]. These late presentations can be attributed to several factors, including lack of awareness, lack of compliance with the screening recommendations, lack of organized regional campaigns for HPV vaccination, and the fact that most health advocacy programs are directed toward other chronic or more prevalent malignancies, such as breast cancer, diabetes, and obesity [11,12,13].

Women in developing countries, especially those with a low socioeconomic status or educational level, are at higher risk of certain health issues, including acquired immunodeficiency syndrome, maternal mortality, and cervical cancer [6]. Although women (≥15 years old) number more than 9.29 million according to the Kingdom’s annual census, the data on the prevalence and awareness of HPV infection and cervical cancer are limited [14]. More than 70% of cervical cancer cases are attributed to high-risk HPV infections [15]. It was found by more than one study that the majority of cases of HPV-related cervical cancer in Saudi Arabia were HPV 16, 18, and 45, which is not different from the international figures [16].

HPV is considered the most important etiological and prognostic factor of CC; therefore, the implementation of early CC screening and HPV vaccination are highly efficacious in decreasing the prevalence and incidence of CC [15]. Regrettably, few studies, with relatively small sample sizes, have been conducted to determine the level of awareness of CC, and women’s acceptance of the HPV vaccine in the different regions of Saudi Arabia. This study aimed to collect data from different regions of Saudi Arabia, and evaluate the national awareness of CC and its risk factors, including HPV infection, and prevention (HPV vaccination) among Saudi females.

## 2. Materials and Methods

This is an observational, cross-sectional, survey-based study that was conducted in Saudi Arabia using an online self-administered questionnaire that was electronically distributed through social media from June to July 2020. All study procedures were performed in accordance with the ethical standards of the Declaration of Helsinki and the universal International Conference on Harmonization-Good Clinical Practice Guidelines. The Institutional Review Board, College of Medicine, King Saud University Hospital approved the study protocol (no. E-20-5069 on 8th September 2020). Written informed consent was obtained from all the participants.

The study included all Saudi women (from different regions of Saudi Arabia) who agreed to participate. Males and non-Saudi women were not included in this study. The sample size was calculated to assess the awareness level of cervical cancer using the Cochran’s Formula n0 = (Z ^2^ pq / e ^2^), in which: Z is the level of confidence (two-sided 95% confidence interval); *p* is the estimated proportion of the population (we used 0.5, which is the maximum variability, as we did not know the exact proportion); q is 1 − p (1 − 0.5 = 0.5); and e is the margin of error, which is 5% (=0.05).

The study’s sample size was expanded to help decrease the risk of sampling errors. The current study included a simple random sample of 564 out of 1692 (33.3%) individuals who fulfilled the study requirements and completed the questionnaire. A pilot study was carried out on seven women to demonstrate the feasibility of the questionnaire, and changes were made according to the results. The final questionnaire contained three main sections with close-ended items. The first part included personal data: age, place of residence, educational level, smoking status, marital status, occupation, monthly income, number of children, and type of contraception used. The second part assessed the participants’ awareness level by asking them whether they had heard the following information before: the presence of cervical cancer, that cervical cancer is treatable if caught early, that cervical cancer is caused by HPV, that HPV can be transmitted sexually, that HPV can cause genital warts, and the existence of an HPV vaccine. In the third part, participants were asked about their source of information. In addition, they were asked whether they had ever had the following: cervical cancer, a cervical cancer screening test, and the HPV vaccine. Finally, the respondents were asked about their willingness to take the HPV vaccine if offered, and to advise their friends and family to take the HPV vaccine. The data were extracted and analyzed using SPSS statics for windows software version 25 (SPSS Inc., Chicago, IL, USA). Categorical variables were expressed as percentages. In order to assess the level of awareness, a score out of 6 was given to each participant based on their answers to the second part of the questionnaire (high scores represent a high level of awareness); after that, multivariable ordinal logistic regression was used for the final score to assess the factors associated with the level of awareness regarding cervical cancer. This study was approved by the King Saud University’s Institutional Review Board. In addition, the participants filled out the online surveys anonymously and voluntarily for the purpose of this study. Furthermore, each participant had a unique IP address to prevent duplications, and the participants were not offered rewards or incentives for participation.

## 3. Results

Among a total of 718 surveys, 564 were completed and unduplicated. The sociodemographic data of the participants are shown in Table 1. The age of the majority of the participating females ranged from 25 to 34 years old (29.8%), followed by 35–44 years (27.1%), 18–24 years old (22.5%), and 55–64 years old (3.2%), and only two (0.4%) participants were younger than 18 years old. The participants’ educational level varied. However, none of them were uneducated. The majority of the participants, accounting for 385 (68.3%), were university graduates. Females with a high school degree represented 115 (20.4%) of the respondents. Furthermore, 36 (6.4%), 10 (1.8%), and 18 (3.2%) participants had a postgraduate degree, secondary-level education, and primary-level education, respectively. Most of the participants were non-smokers (92.2%), married (52.5%), and housewives (63.3%), and had a monthly family income ranging between 10,000 and 20,000 SAR (36.0%). Among the married women, 144 (25.5%) had never used any contraceptive methods. Most of those who opted for contraception used hormonal methods (13.3%), followed by an intrauterine device (IUD) (6.4%), barrier devices (6.2%), or other natural contraceptive methods (5.9%). A slight majority of the participants had children (51.1%).

**Figure 1:**
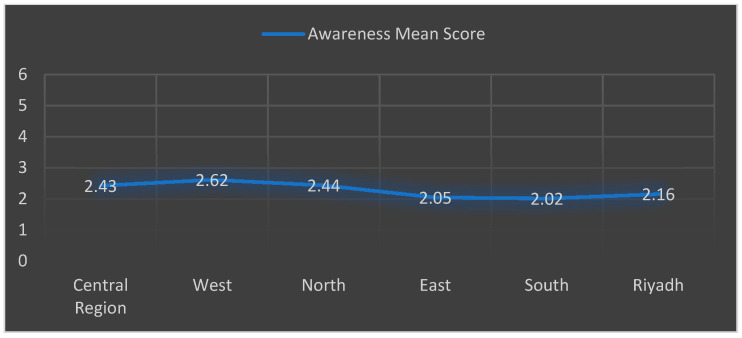
Geographical awareness.

The data collected on the level of awareness are shown in Table 2. A total of 474 (84.0%) of the respondents had heard about cervical cancer, including 348 women (61.7%) who knew that early diagnosis could lead to a better prognosis. In addition, 102 (18.1%) were aware of HPV being a cause of CC, and 119 (21.1%) were knowledgeable about the route of HPV transmission. Furthermore, only 100 females (17.7%) had heard about the HPV vaccine. Lastly, based on the awareness score which was calculated using the six questions in Table 2, we found no difference in the overall level of awareness among the different geographical regions in Saudi Arabia. However, the highest mean score was in the western region (mean score = 2.62/6), and the lowest mean score was in southern region (mean score = 2.02/6), as depicted in Figure 1.

Most of the participants stated that their primary source of information about cervical cancer was the internet (31.4%). Despite these results, only 45 females (8.0%) had a history of cervical screening. Among the 564 participants, only 2 (0.4%) were diagnosed with cervical cancer. By admission, only 11 (2.0%) had taken the vaccine. However, more than half of the respondents (54.1%) were willing to take the vaccine if offered, and most of them (72.9%) were inclined to advise friends and family to take it, as shown in Table 3.

The associations between the sociodemographic variables and the level of awareness are shown in Table 4. The factors that were statistically associated with a higher level of awareness included: age, in which older women were more aware than younger participants (*p*-value < 0.007, 95% CI = 0.071−0.441); high educational level (*p*-value < 0.001, 95% CI = 0.223–0.707); and marital status, in which widows were more aware (*p*-value—0.044, 95% CI = 0.009–0.638).

## 4. Discussion

Despite the fact that most of the participants were educated (95% with a high school education or higher) and had heard about CC (84%), most of them had never had a cervical screening test. These results are in accordance with similar studies conducted in different cities, including Qassim, Jeddah, Riyadh, Hail, and Tabuk [6,13,17,18,19]. These studies have shown a high level of awareness of CC, but a very low rate of cervical screening; for example, those who had a pap smear test in Jeddah formed 16.8% of the total studied population, 15.3% in Qassim, 18% in Hail, and 24.9% in Riyadh [6,8,13,17,19]. Two recent studies attributed the low rate of cervical screening to a lack of knowledge about pap smears; nonetheless, most participants were willing to undergo screening once they were aware of it [8,11]. HPV is a sexually transmitted disease; thus, having multiple sexual partners is one of the main risk factors for HPV infection and subsequent CC. The conservative culture in Saudi makes it challenging to explore this health issue with the required depth, as people do not feel comfortable talking about their sexual life, or they feel that these risk factors or the sexual behaviors leading to HPV do not apply to them, so one would expect that the magnitude of the problem is larger than what is shown in the limited number of local studies. 

Our data showed that 8.6% of the respondents had had relations outside of their current marriage, and we know that a portion of these results refer to previous marriages (6%) or polygamy (6.2%). Unfortunately, most females did not know that HPV is transmitted sexually (78.9%), or that it causes genital warts (81.7%) and CC (81.9%). These results are similar to those published in previous regional studies, which showed that only 14.4% of the general population were aware of HPV as a cause of CC [6]. 

The HPV vaccine has been available since 2006, yet 83.2% of women have never heard about it, and among those who knew about the vaccine, only 11 have taken it (2% of all participants). As per previously published data [6,13,18], more than half of the females were willing to take the vaccine if offered (54.1%), and most of them were willing to advise their friends and family to take it (72.9%). 

Although the literature revealed few studies, conducted in individual hospitals or limited to a certain area of the Kingdom, we found that our cross-sectional data, which included females from all geographical regions, are compatible with the previously published regional studies, and highlight shared aspects [17,19,20]. The level of knowledge of HPV and its clinical implications is poor among Saudi women. There is also a remarkable lack of awareness regarding the HPV vaccine, its importance, and its availability. The main source of information for most Saudi women is the internet, which may be unreliable, or provide misleading information that may delay screening, or discourage vaccination. 

Data from other Arab countries are not far from what we found according to the systematic review done by Rihab Gamaoun. She found that the level of awareness of HPV was low to moderate in all Arab countries. The main obstacles against implementing national programs were mainly due to cultural and religious sensitivities, in addition to the fear of the cost of such vaccinations. Interestingly, acceptability and willingness to get screened or to receive the vaccine was high if the service was free of cost. It was shown in this review that, up to date, the only Arab country which succeeded in implementing a national vaccination program was the United Arab Emirates: their experience showed an absorption rate of 77% in the 1st year, which declined later to 59%. It was blamed on the insufficient media support, and the controversy regarding the side effects of the vaccine [21].

We believe that the current cultural changes in Saudi Arabia which happened over the last twenty years, given that the majority (around 65%) are relatively young and educated people, have helped women to become more confident in discussing such important health issues, and this should lead to more realistic results and a better understanding of HPV status. 

It is known to gynecologic oncologists that cervical cancer patients are relatively young, and present with advanced stages in most cases [22]. It is really impressive to have a vaccine that can prevent more than 90% of cases of such a deadly disease, and, as predicted in large studies, the disease could be eliminated in around three centuries by implementing these programs [23]. The first measure against HPV infection and the subsequent CC is to get vaccinated as early as possible [24]. Due to the low awareness level, the expected resistance from some parents, and given the published experience in many countries when the vaccine was first introduced, the HPV vaccine will not be that easy to establish at a national level in Saudi Arabia [25,26]. The Saudi government, as part of the 2030 vision and strategy, has announced lately that HPV vaccination will be essential for girls in the elementary school (11–14 years old). We believe that this will increase the compliance towards this life-saving vaccine. It was suggested by The Pediatric Pharmacy Advocacy Group (PPAG) and Advisory Council on Immunization Practices (ACIP) in 2007–2008 that the vaccine should be optional up to the parents or the guardians, as the evidence regarding its safety and effectiveness at that time was controversial, waiting for stronger evidence to make it mandatory [27,28]. Making it a compulsory vaccine nowadays after having all this evidence will enable the government to overcome the obstacles of expected resistance. However, that is not an easy task, as in the USA, although HPV was introduced and FDA-approved since 2006, only fifteen out of fifty-one states managed to make it a mandatory school vaccine so far [29].

A very positive report was just published from the United Kingdom showing important and encouraging results of 87% eradication of cervical cancer due to successful implementation of an HPV vaccination program. Such a report should encourage the Saudi government to talk with confidence about its initiative [30].

## 5. Conclusions

Saudi women, like all Arab women, are showing very low awareness about CC prevention, screening, its relation to HPV infection, and the value of anti-HPV vaccination. The government has recently developed a very positive initiative. Scaling-up of this national strategy by the Ministry of Health or other health advocacy agencies is becoming indispensable to overtake the Saudi structural and societal gaps. Countries with no CC screening programs nor HPV vaccination programs should not delay that further, as the value is unquestionable. Our recommendations should be applicable to all Arab countries or any country which is similar in conservative culture and religious beliefs. 

## Figures and Tables

**Table 1:** Sociodemographic data of all participants.

Survey Questions		Number (Total 564)	Percentage (%)
**Age of participants**	<18 years old	2	0.4
	18–24 years old	127	22.5
	25–34 years old	168	29.8
	35–44 years old	153	27.1
	45–54 years old	96	17.0
	55–64 years old	18	3.2
**Level of education**	Primary school	10	1.8
	Secondary school	18	3.2
	High school	115	20.4
	University	385	68.3
	Postgraduate	36	6.4
**Smoking status**	No	520	92.2
	Yes	44	7.8
**Marital status**	Single	221	39.2
	Married	296	52.5
	Widowed	9	1.6
	Divorced	38	6.7
**Occupation**	No	357	63.3
	Yes	207	36.7
**Monthly income**	<5000	93	16.5
	5000–10,000	159	28.2
	10,000–20,000	203	36.0
	20,000–30,000	66	11.7
	>30,000	43	7.6
**Number of children**	No	276	48.9
	Yes	288	51.1
**Type of contraception**	None—not currently married	241	42.7
	None—married	144	25.5
	Hormonal methods	75	13.3
	IUD	36	6.4
	Barrier methods	35	6.2
	Natural contraceptive methods	33	5.9
**Number of marriages**	Never	221	39.2
	1	309	54.8
	2	25	4.4
	3	8	1.4
	4	1	0.2
**Number of husband’s marriages**	Participant is single	221	39.2
	1	307	54.4
	2	28	5.0
	3	7	1.2
	>4	1	0.2
**Number of sexual relations outside current marriage**	None	514	91.1
	1	27	4.8
	2	7	1.2
	3	7	1.2
	4	8	1.4

IUD, intrauterine device.

**Table 2:** Level of awareness regarding cervical cancer and human papillomavirus.

Survey Questions		Number (Total 564)	Percentage (%)
**Participants’ awareness of presence of cervical cancer**	No	90	16.0
	Yes	474	84.0
**Participants’ awareness of the ability to treat cervical cancer if caught early**	No	216	38.3
	Yes	348	61.7
**Participants’ awareness that cervical cancer is caused by HPV**	No	462	81.9
	Yes	102	18.1
**Participants’ awareness that HPV can be transmitted sexually**	No	445	78.9
	Yes	119	21.1
**Participants’ awareness that HPV can cause genital warts**	No	461	81.7
	Yes	103	18.3
**Participants’ awareness about existence of HPV vaccine**	No	464	82.3
	Yes	100	17.7

HPV, human papillomavirus.

**Table 3:** Cervical cancer screening, vaccine status, and source of information.

Survey Questions		Number (Total 564)	Percentage (%)
**Source of information about cervical cancer**	My answer was no	89	15.8
	Internet	272	48.2
	Medical resources	127	22.5
	Family/friends	76	13.5
**Participants who have had cervical cancer**	No	562	99.6
	Yes	2	0.4
**Source of information that cervical cancer can be treated if caught early**	My answer was no	207	36.7
	Internet	177	31.4
	Medical resources	146	25.9
	Family/friends	34	6.0
**Participants who had a cervical cancer screening test**	No	519	92.0
	Yes	45	8.0
**Source of information about HPV vaccine**	My answer was no	451	80.0
	Internet	46	8.2
	Medical resources	59	10.5
	Family/friends	8	1.4
**Participants who took HPV vaccine**	No	553	98.0
	Yes	11	2.0
**Participants who would take HPV vaccine if offered**	No	259	45.9
	Yes	305	54.1
**Participants who would advise friends and family to take HPV vaccine**	No	153	27.1
	Yes	411	72.9

**Table 4:** The statistical association between the variable socioeconomic factors and the level of awareness of cervical cancer.

Variable	Estimate	Std. Error	Wald	*p*-Value	95% Confidence Interval	
					Lower Bound	Upper Bound
**Origin**	−0.064	0.047	1.895	0.169	−0.155	0.027
**Age**	0.256	0.094	7.324	0.007	0.071	0.441
**Education**	0.465	0.123	14.163	<0.001	0.223	0.707
**Smoking**	0.042	0.286	0.022	0.882	−0.518	0.603
**Marital status**	0.324	0.160	4.071	0.044	0.009	0.638
**Occupation**	−0.236	0.175	1.819	0.177	−0.578	0.107
**Monthly income**	0.093	0.061	2.301	0.129	−0.027	0.213
**Number of children**	0.024	0.241	0.010	0.921	−0.448	0.496
**Type of contraception**	0.011	0.063	0.032	0.858	−0.113	0.135

Bold *p*-value represents significance (*p* < 0.05).

## Data Availability

All data are contained within this article.
